# The Causal Effects of Blood Iron and Copper on Lipid Metabolism Diseases: Evidence from Phenome-Wide Mendelian Randomization Study

**DOI:** 10.3390/nu12103174

**Published:** 2020-10-17

**Authors:** Jingqi Zhou, Chang Liu, Michael Francis, Yitang Sun, Moon-Suhn Ryu, Arthur Grider, Kaixiong Ye

**Affiliations:** 1 Department of Genetics, University of Georgia, Athens, GA 30602, USA; jingqi.zhou@uga.edu (J.Z.); chang.liu1@uga.edu (C.L.); yitang.sun@uga.edu (Y.S.); 2 School of Public Health, Shanghai Jiao Tong University School of Medicine, Shanghai 200025, China; 3 College of Life Sciences, Wuhan University, Wuhan 430072, China; 4 Institute of Bioinformatics, University of Georgia, Athens, GA 30602, USA; michaelfrancis@uga.edu; 5 Department of Food Science and Nutrition, University of Minnesota, St. Paul, MN 55108, USA; mryu@umn.edu; 6 Department of Foods and Nutrition, University of Georgia, Athens, GA 30602, USA; agrider1@uga.edu

**Keywords:** iron, copper, phenome-wide association study, Mendelian randomization, lipid metabolism disorder

## Abstract

Blood levels of iron and copper, even within their normal ranges, have been associated with a wide range of clinical outcomes. The available epidemiological evidence for these associations is often inconsistent and suffers from confounding and reverse causation. This study aims to examine the causal clinical effects of blood iron and copper with Mendelian randomization (MR) analyses. Genetic instruments for the blood levels of iron and copper were curated from existing genome-wide association studies. Candidate clinical outcomes were identified based on a phenome-wide association study (PheWAS) between these genetic instruments and a wide range of phenotypes in 310,999 unrelated individuals of European ancestry from the UK Biobank. All signals passing stringent correction for multiple testing were followed by MR analyses, with replication in independent data sources where possible. We found that genetically predicted higher blood levels of iron and copper are both associated with lower risks of iron deficiency anemia (odds ratio (OR) = 0.75, 95% confidence interval (CI): 0.67–0.85, *p* = 1.90 × 10^−6^ for iron; OR = 0.88, 95% CI: 0.78–0.98, *p* = 0.032 for copper), lipid metabolism disorders, and its two subcategories, hyperlipidemia (OR = 0.90, 95% CI: 0.85–0.96, *p* = 6.44 × 10^−4^; OR = 0.92, 95% CI: 0.87–0.98, *p* = 5.51 × 10^−3^) and hypercholesterolemia (OR = 0.90, 95% CI: 0.84–0.95, *p* = 5.34 × 10^−4^; OR = 0.93, 95% CI: 0.89–0.99, *p* = 0.022). Consistently, they are also associated with lower blood levels of total cholesterol and low-density lipoprotein cholesterol. Multiple sensitivity tests were applied to assess the presence of pleiotropy and the robustness of causal estimates. Regardless of the approaches, consistent evidence was obtained. Moreover, the unique clinical effects of each blood mineral were identified. Notably, genetically predicated higher blood iron is associated with an enhanced risk of varicose veins (OR = 1.28, 95% CI: 1.15–1.42, *p* = 4.34 × 10^−6^), while blood copper is positively associated with the risk of osteoarthrosis (OR = 1.07, 95% CI: 1.02–1.13, *p* = 0.010). Sex-stratified MR analysis further revealed some degree of sex differences in their clinical effects. Our comparative PheWAS-MR study of iron and copper comprehensively characterized their shared and unique clinical effects, highlighting their potential causal roles in hyperlipidemia and hypercholesterolemia. Given the modifiable nature of blood mineral status and the potential for clinical intervention, these findings warrant further investigation.

## 1. Introduction

Iron (Fe) and copper (Cu) are two essential mineral nutrients for human health through their vital roles in enzymatic reactions and cellular energy metabolism [1,2]. Physiological processes that maintain the homeostasis of these minerals are often influenced by dietary loss, malabsorption, inflammation, infection, liver disease, and dysregulated erythropoiesis. Impaired systemic iron or copper homeostasis, including deficiency, excess, and even fluctuations in the normal ranges, could have clinical implications [3]. Iron deficiency, the most widespread micronutrient deficiency worldwide, is well-established to cause anemia, while iron overload can lead to chronic liver disease, cirrhosis, and hepatocellular carcinoma [4,5]. Copper is a cofactor of many redox enzymes, and it participates in iron metabolism either by competing with iron for binding ligands or through the action of various iron-regulating cuproenzymes [6]. Copper is protective against iron deficiency anemia, and the mechanistic basis is relatively well-established [7]. First, the efflux of iron from enterocytes, hepatocytes, and macrophages into the bloodstream requires the action of two copper-dependent ferroxidases (i.e., hephaestin and ceruloplasmin), which oxidize ferrous iron into the transferrin-binding ferric iron [8]. In addition to enabling iron transport, copper is also required for hemoglobin biosynthesis, probably by assisting iron import into or utilization in mitochondria [9]. These interactions of copper and iron likely underlie their shared associations with various diseases. For instance, epidemiological studies have reported that elevated blood levels of iron and copper are associated with a higher risk of type 2 diabetes, anemia, and osteoarthritis [10,11]. Serum ferritin and plasma copper have been associated with an improved blood lipid profile, corresponding to a reduced risk of hyperlipidemia [12,13]. However, conflicting associative patterns have also been reported [14]. Since most existing studies were observational and often complicated by reverse causality and residual confounding, it is still unknown whether these associations indicate causal relationships.

Mendelian randomization (MR), a complementary approach to epidemiological observations, utilizes genetic variants as instrumental variables to approximate the lifetime status of an exposure (e.g., the blood level of a mineral) and evaluates its causal effect on a clinical outcome. The random allocation of alleles at conception and the natural direction of causality from genetic variants to phenotypes protect MR estimates from confounding and reverse causality [15,16]. MR studies have been performed to evaluate the causality of specific mineral–outcome pairs, such as iron on stroke, coronary artery disease, and Parkinson’s disease [17,18,19]; and copper on ischemic heart disease [20]. Compared to these hypothesis-driven MR studies with an obvious bias toward cardiovascular diseases, a phenome-wide association study coupled with MR (PheWAS-MR) enables an unbiased and hypothesis-free scan through a wide range of phenotypes (i.e., phenome) and prioritized candidate clinical outcomes for MR causal inferences. PheWAS-MR has been conducted on iron, but not copper [21]. Most importantly, few existing studies simultaneously examine multiple blood minerals at a phenome-wide scale to disentangle their confounded clinical effects [22].

In this study, we systematically evaluated and compared the causal clinical effects of iron and copper. Genetic instruments for the blood levels of iron and copper were curated from existing genome-wide association studies (GWAS) with the largest sample sizes. Candidate clinical outcomes were identified based on a phenome-wide association study between these genetic instruments and a large number of disease outcomes from the UK Biobank (UKBB). All signals passing stringent correction for multiple testing were followed by MR analyses. Additionally, we examined the causal association between the iron or copper level and a subset of lipid profiles, which are essential biomarkers for lipid metabolism diseases, in a secondary analysis with an independent dataset.

## 2. Methods

**Figure 1:**
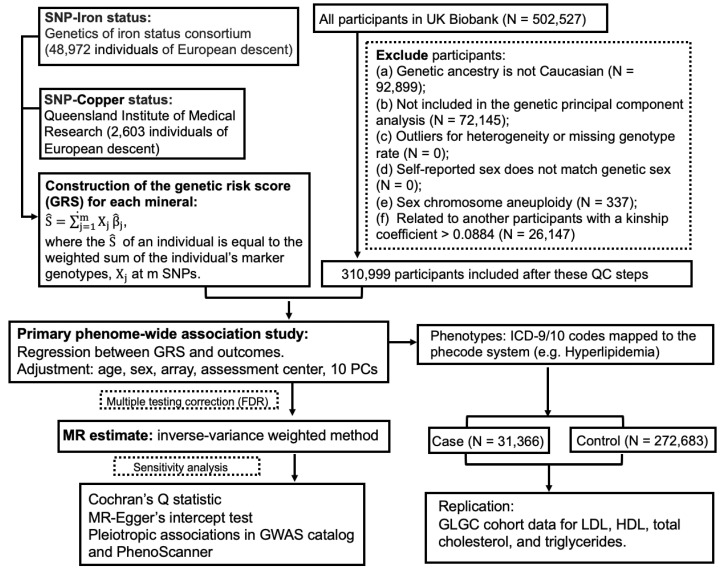
A flow chart of the study design.

The guideline of Strengthening the Reporting of Observational Studies in Epidemiology (STROBE) (Table S1) was followed in this study report. The UKBB project was approved by the North West Multi-Centre Research Ethics Committee (11/NW/0382), and appropriate informed consent was obtained from participants. Data used in the project were accessed through an approved application to UKBB. Only a subset of unrelated white British individuals with high-quality genotype data (*N* = 310,999) were included in this study in order to minimize the complication of population structure. The study design of PheWAS-MR, replication, and sensitivity analysis was prespecified. A schematic of the overall study design is shown in Figure 1.

### 2.1. Genetic Instruments for Blood Iron and Copper

Independent genetic instrumental variables for the blood levels of iron and copper were selected from previous GWAS (Table 1). Based on a meta-analysis of 48,972 subjects of European descent performed by the Genetics of Iron Status consortium [23], three single nucleotide polymorphisms (SNPs) were selected as genetic instruments for systemic iron status. These three SNPs are consistently associated with four iron status biomarkers, including serum iron, ferritin, transferrin, and transferrin saturation. Their effects on serum iron were used in the MR analysis. They are strong genetic instruments, with F statistics ranging from 445 to 796 and collectively explaining approximately 3.80% of the variation in serum iron. They have been previously used in MR analyses of iron status [18]. Genetic variants associated with the erythrocyte copper level were identified in an Australian cohort (*N* = 2603) [24]. Two SNPs independently associated with erythrocyte copper were selected, which accounted for 4.60% of the phenotypic variance. For each blood mineral, a weighted polygenic risk score (GRS) was constructed for each UKBB participant by summing over selected SNPs weighted by their effect sizes.

### 2.2. Study Population

The UKBB is a population-based prospective cohort established to study genetic and environmental determinants of human diseases. More than 500,000 individuals aged 40–69 years were recruited between 2006 and 2010, all of whom gave written consent and underwent baseline measurements. Participants donated blood samples for genotyping and biomarker analysis. Moreover, UKBB participants consented access to their electronic health records. Only participants fulfilling the following criteria were included in our analysis: genetic ancestry is European, included in genetic principal component analysis, not outliers for heterogeneity or missing genotype rate, no sex chromosome aneuploidy, no high degree of genetic kinship (i.e., ten or more third-degree relatives identified), and self-reported gender matching genetic sex. Additionally, for the remaining pairs of relatives (kinship coefficient >0.0884), a minimum number of participants were removed so that all remaining participants are unrelated. Analysis was restricted to participants of European descent in order to maintain consistency with the European samples used to obtain genetic instruments for blood mineral status. A total of 310,999 unrelated individuals were included in our final analysis. Of these participants, 53.5% were female, and the mean age was 56.86 (SD 8.0) years at baseline.

### 2.3. Phenome-Wide Association Study

Three phenotypic datasets in the UKBB, including inpatient hospital records, cancer registry, and death registry, were included in this analysis. First, we used the International Classification of Diseases (ICD) versions 9 and 10 to identify cases in the hospital episode statistics, including both incident and prevalent cases, but not self-reported diagnoses. As ICD-9/ICD-10 codes are not organized as independent phenotypes, we mapped them to the phecode system of distinct diseases or traits [25]. The mapping process also automatically excluded patients with similar or potentially overlapping diseases from the corresponding control group. The online phecode map is accessible via the link https://phewascatalog.org/phecodes_icd10. We restricted analysis to phecodes with sufficient numbers of cases to ensure more than 80% statistical power in subsequent MR analyses. The basic statistics of phenotypes and their case numbers are shown in Table S2. The phecode 272.1 was used for the identification of hyperlipidemia, and the phecode 272.11 was used for the identification of hypercholesterolemia. For each mineral, case-control groups were generated for each phecode, and logistic regression was performed separately for each instrument SNP, adjusting for age, sex, genotyping array, assessment center, and the first 10 genetic principal components. Moreover, we explored the association between the weighted GRS of each blood mineral and each phecode with logistic regression. Given that phenotypes investigated are not totally independent even in the phecode system, we applied the false discovery rate (FDR) method with a cutoff of 0.05 to correct for multiple testing.

### 2.4. MR Analyses

To assess whether blood mineral levels have causal effects on the candidate clinical outcomes identified by the PheWAS, we further conducted two-sample MR analyses, where the instrument–mineral and instrument–outcome associations were estimated in two different samples. In this study, the instrument–mineral effect was estimated in previous GWAS, while the instrument–outcome effect was estimated in the above-mentioned PheWAS. To estimate the causal effect of each mineral on a phenotype, for each genetic instrument, we calculated the ratio of its effect on the phenotype to its effect on the blood mineral. An overall effect estimate across multiple genetic instruments was achieved with three methods, the inverse-variance weighted (IVW) method, weighted median (WM) method, and MR-Egger [26,27]. Effect estimates, measured as the odds radio (OR) of the outcome, were normalized to one standard deviation (SD) increment in each blood mineral. Additionally, sex-stratified IVW MR estimates were obtained with sex-specific instrument–outcome effects from genetically males and females, respectively. Mineral–outcome pairs exhibiting evidence of horizontal pleiotropy were excluded in this stratified analysis.

To evaluate the effects of blood iron and copper on four blood lipids, including total cholesterol (TC), high-density lipoprotein cholesterol (HDL cholesterol), low-density lipoprotein cholesterol (LDL cholesterol), and triglycerides, we first performed MR analysis with the instrument–outcome associations estimated in the UKBB. To validate the mineral–outcome relationships that we discovered based on the UKBB, we performed replication analysis in a different cohort, the Global Lipids Genetics Consortium (GLGC). The instrument–outcome summary statistics from this GWAS were accessed through the MR-Base [28,29]. To further evaluate if the effects of two minerals on a shared clinical outcome are independent of each other, we adopted a multivariable MR framework [30], in which the effects of multiple related risk factors can be estimated simultaneously. For instance, to test if the effects of iron and copper on a shared clinical outcome (e.g., hyperlipidemia) are independent, we included all the genetic instruments for iron and copper in the analysis. The effect sizes of instrument SNPs on blood iron and copper were extracted from previous GWAS [23,24], while their effects on the shared clinical outcome were calculated in our curated UKBB dataset. We modeled the SNP effects on the shared outcome (β_shared_) against the SNP effects on iron (β_Fe_), adjusted for the effects on copper (β_Cu_), using a weighted linear regression model (β_shared_~β_Fe_ + β_Cu_), where the weights were defined by inverse standard errors of β_shared_.

### 2.5. Sensitivity Analyses

We first evaluated the strength of the genetic instruments with the F statistic, and the degree of violation of the NO Measurement Error (NOME) assumption using the I^2^ statistic [31]. In cases where the NOME assumption is violated, the simulation extrapolation (SIMEX) method was used to correct for attenuation bias [31]. Several sensitivity analyses were further performed to detect and correct for the presence of pleiotropy in the causal estimates. Since the IVW estimate is essentially a weighted average of the Wald ratios obtained from each SNP, if any of the SNPs exhibit horizontal pleiotropy, then the causal effect estimate is liable to be biased. We obtained the IVW estimate by integrating the SNP-specific Wald estimates using the multiplicative random-effects model. The possible presence of heterogeneity across genetic instruments were evaluated with Cochran’s Q test. We further performed WM MR [27] and MR-Egger analyses [32]. The WM analysis calculates the median of an empirical distribution of MR association estimates weighted by their precisions. It provides consistent estimates when more than half of the instruments are valid [27]. The MR-Egger method provides an intercept test, with a non-zero intercept indicating the presence of unbalanced horizontal pleiotropy. It also provides an unbiased estimate of the causal effect, taking into account pleiotropy. In addition to the above-mentioned sensitivity analyses that leverage information from multiple genetic instruments for one blood mineral, for each individual genetic instrument, we also evaluated their pleiotropic effects by searching the GWAS catalog and PhenoScanner V2 [33], to identify any secondary phenotypes associated (*p* < 5 × 10^−8^) with them or their proxies (r^2^ > 0.8). Lastly, we performed a leave-one-out analysis to confirm that the results are not driven by a specific genetic instrument.

All analyses were conducted using the MendelianRandomisation [34] and TwoSampleMR [28] packages, and the R programming language.

## 3. Results

After quality controls on the full UKBB dataset, a subset of 310,999 unrelated individuals of European descent were included in the PheWAS analysis. The main demographic characteristics of the study population and basic statistics of all genetic instruments are summarized in Table S3. All instrument SNPs satisfied the Hardy–Weinberg equilibrium test (*p* > 0.05). The association between the weighted GRS of each blood mineral and common confounding factors (i.e., age, sex, genotyping array, assessment center, and the genetic principal components) are presented in Table S4. All these factors were controlled for in the PheWAS analysis.

PheWAS analysis was limited to phenotypes (i.e., phecodes) that had enough cases to ensure more than 80% statistical power in the subsequent MR analyses. Among them, 12 clinical outcomes, representing six disease categories, reached statistical significance at the 5% FDR threshold. Next, we performed MR analyses to examine the possible causal links of blood iron and copper to each of the significant outcomes identified in PheWAS. Mineral–outcome pairs exhibiting consistent MR evidence for causal effects, without suggestions of heterogeneity or horizontal pleiotropy across genetic instruments based on Cochran’s Q statistic and MR-Egger’s intercept test, are presented here (Table S5).

### 3.1. Shared and Unique Causal Clinical Effects of Blood Iron and Copper

**Figure 2:**
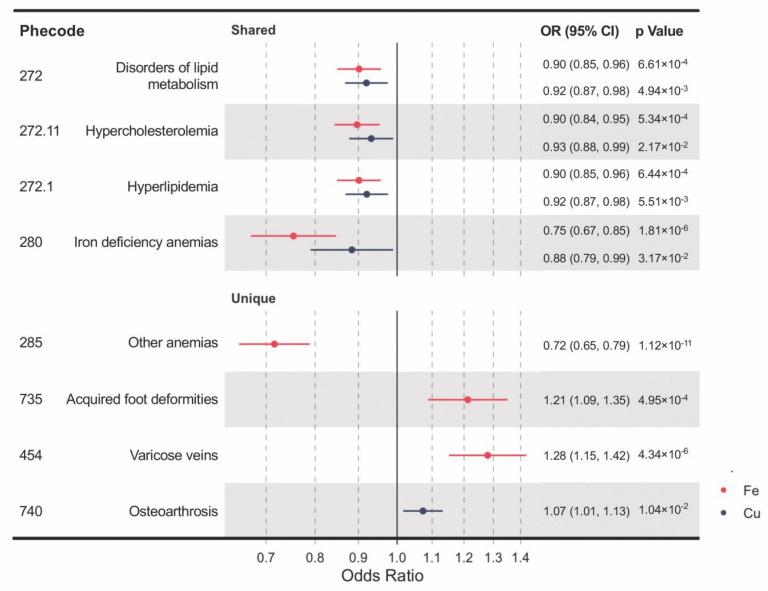
A forest plot showing significant mineral–outcome associations based on MR analysis. The causal estimates are from inverse-variance weighted (IVW) MR and have no indications of pleiotropy. The odds ratios (ORs) with their 95% confidence intervals (CIs) are scaled to a 1-SD increase in blood iron or copper level. Complete MR results are provided in Table S5.

Genetically determined blood levels of iron and copper share some of the associated clinical outcomes, including iron deficiency anemia, disorders of lipid metabolism, hypercholesterolemia, and hyperlipidemia (Figure 2). IVW MR analyses provided significant evidence for causal effects of both higher blood iron and copper on reduced risks of iron deficiency anemia (OR per SD increase in blood iron = 0.75, 95% confidence interval (CI): 0.67–0.85, *p* = 1.81 × 10^−6^; OR per SD increase in blood copper = 0.88, 95% CI: 0.79–0.99, *p* = 0.032) and disorders of lipid metabolism (OR = 0.90, 95% CI: 0.85–0.96, *p* = 6.61 × 10^−4^; OR = 0.92, 95% CI: 0.87–0.98, *p* = 4.94 × 10^−3^), and its two subtypes, hyperlipidemia (OR = 0.90, CI: 0.85–0.96, *p* = 6.44 × 10^−4^; OR = 0.92, CI: 0.87–0.98, *p* = 5.51 × 10^−3^) and hypercholesterolemia (OR = 0.90, CI: 0.84–0.95, *p* = 5.34 × 10^−4^; OR = 0.93, CI: 0.88–0.99, *p* = 0.022). Moreover, unique causal clinical effects were identified for iron and copper. Per SD increase in blood iron is associated with enhanced risks of varicose veins (OR = 1.28, CI: 1.15–1.42, *p* = 4.34 × 10^−6^) and acquired foot deformities (OR = 1.21, CI: 1.09–1.35, *p* = 4.95 × 10^−4^) but with reduced risk of other anemia (OR = 0.72; CI: 0.65–0.79; *p* = 1.12 × 10^−11^). Per SD increment in blood copper is associated with an increased risk of osteoarthrosis (OR = 1.07, CI: 1.02–1.13, *p* = 0.010).

### 3.2. Causality of Both Iron and Copper on Lipid Metabolism Traits

We performed a further investigation into the shared clinical effects of blood iron and copper on lipid metabolism disorders, by first evaluating if they are independent of each other and then by examining their causal effects on blood biomarkers of lipid metabolism, including TC, HDL cholesterol, LDL cholesterol, and triglycerides. First, we adopted a multivariable MR framework to estimate the direct effects of blood iron and copper on their shared clinical outcomes by conditioning on the effects of the other mineral. We found that the causal effects of iron and copper on lipid metabolism disorders are independent of each other and do not show substantial differences from univariate MR estimates (Table S6).

Based on the shared but independent effects of iron and copper on lipid metabolism disorders, we further hypothesized that their genetically predicted levels are associated with blood biomarkers of lipid metabolism. We tested this hypothesis in the UKBB with the exclusion of participants under statin medication and in an independent replication cohort from the GLGC (Table 2). Per SD increment of blood iron is associated with an 0.089 mmol/L decrease of LDL cholesterol (WM β = −0.089, SE = 0.014, *p* = 5.27 × 10^−11^ in UKBB) and 0.096 mmol/L decrease of TC (WM β = −0.096, SE = 0.018, *p* = 8.99 × 10^−8^ in UKBB) but an 0.047 mmol/L increase of triglycerides (WM β = 0.047, SE = 0.012, *p* = 4.2 × 10^−5^ in UKBB). While the IVW method yields significant results in the same effect directions, horizontal pleiotropy was detected. WM estimates are robust to pleiotropy as long as more than half of the genetic variants are valid instruments. In GCLC, the same effect directions with statistical significance were observed with the WM method. No significant effects were found for HDL cholesterol in either cohort. For blood copper, while no significant effects were found in the UKBB, analysis in the GCLC cohort showed that per SD increase in copper is associated with a 0.048 mmol/L reduction of LDL cholesterol (IVW β = −0.048, SE = 0.019, *p* = 0.011 in GCLC) and 0.043 mmol/L reduction of TC (IVW β = −0.043, SE = 0.018, *p* = 0.02 in GCLC). Overall, we found evidence for shared effects of iron and copper in reducing the blood levels of LDL cholesterol and TC.

### 3.3. Interpretation of Potential Pleiotropy in MR Results

To detect and correct for any possible pleiotropic effect of multiple instruments, we conducted multiple sensitivity analyses. First, F statistics for all genetic instruments were >10, indicating that weak instrument bias was unlikely to affect the IVW analyses. With regard to the potential violation of the NOME assumption, I^2^_GX_ statistics indicated that measurement errors in the SNP–exposure associations do not substantially attenuate the exposure to outcome effect estimates (I^2^_GX_ > 0.9). All causal clinical effects of iron and copper presented above in the combined analysis do not have evidence for heterogeneity or horizontal pleiotropy across genetic instruments, as indicated by Cochran’s Q statistic and MR-Egger’s intercept test (Table S5). The MR estimates with the SIMEX adjustment were directionally consistent with the unadjusted estimates (Table S5). Leave-one-out analysis for each candidate phenotype did not alter the results substantially (Table S7). For some relationships, causal estimates from WM MR and MR-Egger are broadly consistent with those from IVW MR, although with wider confidence intervals as expected from their lower statistical powers [35]. For instance, the causal estimates of blood iron on disorders of lipid metabolism were consistent in the IVW MR (OR = 0.90, CI: 0.85–0.96, *p* = 6.61 × 10^−4^) and WM MR (OR = 0.91, 95% CI: 0.85–0.97, *p* = 6.25 × 10^−3^). In addition to sensitivity analyses that leverage information from multiple genetic instruments for one blood mineral, for each individual instrument SNP, we also evaluated its pleiotropic effects by searching the GWAS catalog and PhenoScanner V2 [33]. The *HFE* rs1800562 variant is associated with LDL cholesterol [29], and this SNP was identified as potentially pleiotropic, although it likely represents vertical pleiotropy in the analysis of lipid-related traits and does not violate the MR assumptions. Similar magnitudes of associations were observed when this variant was excluded from the MR analyses (Table S7).

**Figure 3:**
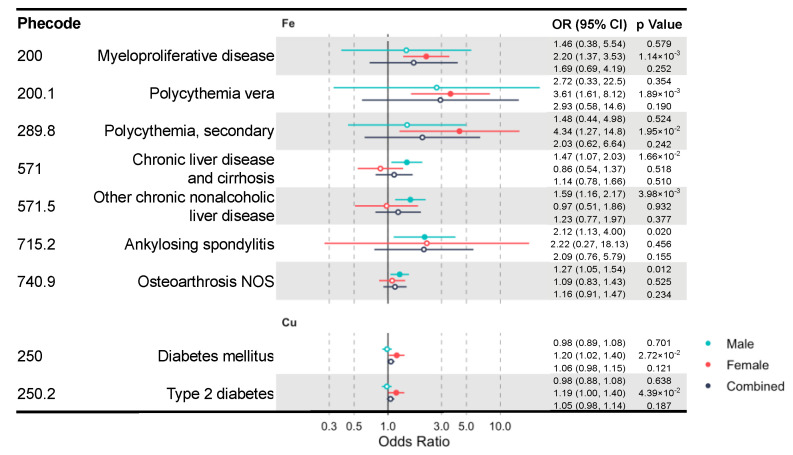
A forest plot showing significant mineral–outcome associations based on sex-stratified MR analysis. Results in the male, female, and combined analyses are shown. The causal estimates are from IVW MR and have no indications of pleiotropy. The odds ratios (ORs) with their 95% confidence intervals (CIs) are scaled to a 1-SD increase in blood mineral level. Complete MR results are provided in Table S8.

Due to the well-known sex differences in iron and copper status and metabolism [36,37,38,39], we additionally performed sex-stratified IVW MR analysis to identify sex-specific clinical effects of these minerals. We identified multiple mineral–outcome pairs exhibiting possible sex-specific causal relationships (Figure 3, Table S8). That is, their MR estimates are significant only in one sex group, but not in the other or the sex-combined sample. Iron is associated with higher risks of myeloproliferative diseases (OR = 2.20, CI: 1.37–3.53, *p* = 1.14 × 10^−3^) in females and chronic liver disease (OR = 1.47, CI: 1.07–2.03, *p* = 0.017) in males. Moreover, copper increases the risk of diabetes mellitus (OR = 1.20, CI: 1.02–1.40, *p* = 0.027) and its subcategory, type 2 diabetes (T2D) (OR = 1.19, CI: 1.00–1.40, *p* = 0.044) in females. However, blood iron and copper have no sex-specific effects on lipid metabolism diseases.

## 4. Discussion

Our study adopted a phenome-wide approach to systematically evaluate and compare the clinical effects of blood iron and copper. This is the first PheWAS-MR study for copper, and the first to perform systematic comparison across iron and copper. The MR approach reduces the biases from confounding and reverse causality, which affect most observational associations. This strategy utilizes genotype–exposure and genotype–disease associations to strengthen inferences between modifiable exposure and diseases, aiming to reduce disease risk in the population by modifying the exposure through lifestyle changes or clinical interventions. Our findings highlight the shared protective effects of iron and copper on lipid metabolism disorders and iron deficiency anemia. Some potential causal mineral–outcome relationships identified in this study have been well known and supported by existing mechanistic studies, while others are novel, awaiting further confirmation and future mechanistic exploration.

Most notably, we found that genetically predicted higher blood levels of iron and copper are both protective against lipid metabolism disorders and its two subcategories, hyperlipidemia and hypercholesterolemia. Consistently, they are also associated with lower blood TC and LDL cholesterol levels. This is the first MR study to establish the probable causal protective effect of copper on lipid metabolism disorders. The interplays among lipid, iron, and copper metabolism have been recognized, but they are complex and not fully elucidated. Epidemiological findings of the effect of copper on lipid metabolism are equivocal, with some studies showing negative associations of blood copper with both TC and LDL cholesterol [40], and others showing positive associations [41]. The relationship between iron status and blood lipids is similarly ambiguous. Some epidemiological studies found that blood iron is lower in hyperlipidemic patients [42] and that blood ferritin is negatively associated with LDL cholesterol [13]. Very likely related, elevated iron status is negatively associated with coronary artery disease in both observational and MR studies [43,44]. Other studies have found that anemic and/or iron-deficient patients and animal models tend to have lower blood TC and LDL cholesterol [45], while blood ferritin has often been found to be positively associated with an unhealthy lipid profile (i.e., elevated TC, LDL cholesterol, and triglyceride, but decreased HDL cholesterol) [46]. These conflicting observations likely suffer from residual confounding and reverse causation, highlighting the importance of MR studies.

To gain mechanistic insights into how iron regulates lipid metabolism, we searched for iron-responsive elements in genes involved in cholesterol metabolism and fatty acid degradation using available databases [47]. Multiple potential iron-responsive genes were identified, including *LIPH* and *LDLRAP1* (Figures S1 and S2). Additional mechanistic insights into the potential protective effects of blood iron on lipid metabolism disorders can be drawn from studies on rats. It has been shown that iron deficiency upregulates lipogenic genes but downregulates apolipoprotein H and genes involved in the mitochondrial beta-oxidation, resulting in increased circulating lipids [48]. For copper, its deficiency has long been linked to increased risks of hyperlipidemia and cardiovascular diseases in both humans and animal models [49], while copper supplementation in patients of hyperlipidemia was shown to improve the blood lipid profile [50]. Multiple possible molecular mechanisms have been suggested for the effect of copper deficiency on lipid metabolism disorders, mainly from studies on copper-deficient rats. First, copper deficiency has been shown to increase the level of a key enzyme in the cholesterol synthesis pathway, hydroxymethylglutaryl-coenzyme A (HMG-CoA) reductase [49,51]. An intestinal transcriptome analysis found that in copper-deficient rats, genes involved in mitochondrial and peroxisomal fatty acids beta-oxidation are down-regulated, and genes involved in plasma cholesterol transport are up-regulated [52]. It was also observed that HDL apolipoprotein catabolism was increased, but HDL uptake did not change in the liver and adrenal gland, which are organs that can further metabolize cholesterol [53,54]. Our study highlights the deficiency of iron and copper as likely causal risk factors for dyslipidemia and calls for future studies into their physiological mechanisms.

Well-known mineral–outcome relationships with established physiological mechanisms provide support for the power and validity of our study. Iron is an integral component of the oxygen-carrying hemoglobin and is required for the process of erythropoiesis [4]. In agreement with this, we found that a genetically predicted higher level of blood iron is protective against anemia, while in women, it increases the risks of myeloproliferative disease, polycythemia vera, and secondary polycythemia. Myeloproliferative diseases are bone marrow and blood disorders featured by abnormal hematopoiesis, while its most common subtype, polycythemia vera, is characterized by erythrocytosis (i.e., excessive red blood cell production) [55]. Very interestingly, iron deficiency without anemia is present in virtually all patients of polycythemia vera [56]. Our findings suggest that iron deficiency is the result, rather than the cause, of erythrocytosis and reaffirm the current practice of not using iron supplements to treat patients of polycythemia vera. At the other extreme, iron overload can cause tissue damage, and its excessive accumulation in the liver leads to cirrhosis and hepatocellular carcinoma [3]. Consistently, our study revealed that elevated blood iron in men is positively associated with risks of chronic liver disease and cirrhosis, and one of its subtypes, other chronic nonalcoholic liver disease.

Our study revealed a novel role of blood iron in increasing the risk of varicose veins. Varicose veins, a common venous disease of the lower extremity, is characterized by incompetent valves, reflux, and venous wall dilation. Its etiological process involves the hydrostatic-pressure-induced activation of matrix-degrading enzymes and inflammatory cascade [57]. Iron overload and its causal *HFE* genetic variations have been associated with the development of varicose veins [58]. Mechanistically, iron overload induces oxidative stress and the hyperactivation of matrix-degrading enzymes [59]. Other significant mineral–outcome relationships are also worth mentioning. We found that genetically predicted higher blood iron is associated with an increased risk of glossitis (i.e., tongue inflammation), which is consistent with what has been reported in a previous MR study [21]. However, it is fairly well established that patients of atrophic glossitis frequently suffer from deficiencies of nutrients, including iron, and corresponding nutrient supplementation is able to resolve oral symptoms [60]. The reconciliation of these disparate data is needed in future studies. Our findings open many new research avenues to elucidate the roles of blood iron and copper in these clinical conditions.

Our study has strengths and limitations. This is the first comparative PheWAS-MR of iron and copper, based on a large prospective cohort, to simultaneously evaluate their shared and unique causal clinical effects. Our approach is unbiased and hypothesis-free. In this study, only MR estimates with no indications of pleiotropy were reported. For selected outcomes (i.e., blood lipids), we replicated the results in an independent dataset. The sex-stratified analysis enabled the identification of sex-specific relationships. Our results confirmed some previous MR findings while making novel discoveries, most of which are supported by existing epidemiological and mechanistic studies. Our study has a number of limitations. The first assumption of MR is that the genetic instrument must be strongly associated with the exposure [61]. We attempted to satisfy this assumption by using exposure-associated genetic variants at the genome-wide significance level. However, these instruments only explain a small portion of the phenotypic variance of these blood minerals. The instrumental variables we used explain ≈3.8% of the variance in blood iron status and 4.6% for blood copper in relatively small samples. This means that the analyses may be subject to weak instrument bias, which may reduce the statistical power to identify significant associations [62]. However, our F and I^2^_GX_ statistics for all instruments suggest that our results were not substantially affected by weak instrument bias. Nevertheless, it would be essential to repeat these analyses using instruments from better powered GWAS with larger sample sizes. Additionally, we could not fully rule out the possibility that horizontal pleiotropy affected our results. Although the presence of horizontal pleiotropy can be examined or corrected using Cochran’s Q test, the MR-Egger tests, and WM MR, these methods usually require a large number of instrument SNPs [63]. Without a large enough number of genetic instruments SNPs, some of our results need to be interpreted with caution. Still, we want to emphasize that our leave-one-out sensitivity analysis confirmed that our results were not driven by any specific variants.

Despite a large sample size (*N* = 310,999) of the overall cohort, the case numbers of specific outcomes are still small, limiting our statistical power for rare diseases and those with modest effects. Small sample sizes may also play a role in our sex-stratified analysis, with reduced statistical power in the male and female groups, compared to the combined group. It is possible that some negative results in one gender group are due to a lack of power. However, we focused on outcomes that are significant in one gender group but not significant in either the other gender group or the combined group. If the effects are consistent across the genders, they should have been detected in the combined analysis. Another related issue is that MR estimation assumes a linear relationship between the exposure and the outcome, and as a result, non-linear effects might have been missed. Given the well-known threshold effects of mineral deficiency and overload, future studies with a non-linear model will likely reveal more clinical effects. Genetic instruments approximate the average effects over the life course, while the physiological relevance of a blood mineral could vary by life stages and is not captured by our study. Finally, the UKBB is a middle-and old-aged cohort, and we restricted the analysis to those of European descent. Future studies in cohorts of younger ages or other ethnic backgrounds are needed to confirm our findings and to search for more clinical consequences.

## 5. Conclusions

Our comparative PheWAS-MR study of blood iron and copper comprehensively characterized their shared and unique clinical effects. These known and novel mineral–outcome relationships provide profound insights into the health impacts of normally varying blood minerals and into the etiologies of some clinical conditions, especially hyperlipidemia and hypercholesterolemia. Our findings also emphasize the possibility and importance of managing blood minerals, probably through dietary adjustment, in the prevention and management of these medical conditions.

## Figures and Tables

**Table 1:** Single nucleotide polymorphisms (SNPs) used as genetic instruments for blood minerals in the phenome-wide association study (PheWAS) and Mendelian randomization (MR) analyses.

SNP	Effect Allele	Baseline Allele	Chr	Closest Gene	% Variance Explained	F-Statistic	EAF	Beta ^a^	SE	P
3 SNPs for Fe from Benyamin et al. (*N* ^b^ = 48,972)										
rs1800562	A	G	6	*HFE*	1.30	645	0.067	0.328	0.016	2.72 × 10^−97^
rs1799945	G	C	6	*HFE*	0.90	445	0.15	0.189	0.010	1.10 × 10^−81^
rs855791	G	A	22	*TMPRSS6*	1.60	796	0.554	0.181	0.007	1.32 × 10^−139^
2 SNPs for Cu from Evans et al. (*N* ^b^ = 2603)										
rs1175550	G	A	1	*SMIM1*	1.45	38	0.23	0.198	0.032	5.03 × 10^−10^
rs2769264	G	T	1	*SELENBP1*	3.15	85	0.18	0.313	0.034	2.63 × 10^−20^

SNP: single nucleotide polymorphism, Chr: chromosome, EAF: effect allele frequency, SE: standard error. ^a^. The beta coefficient of mineral-increasing allele on concentrations of blood iron (in μmol/L), blood copper (in mmol/L). ^b^. The sample size of the genome-wide association studies (GWAS) or meta-analysis from which the genetic variants were selected.

**Table 2:** MR analyses of iron and copper on blood lipids (mmol/L) in UK Biobank (UKBB) and Global Lipids Genetics Consortium (GLGC).

Exposure/Outcome	MR Method	Beta	SE	95% CI	P-Effect	P-Pleiotropy	*n*_Total	Data
Iron								
HDL cholesterol	WM	−0.008	0.015	(−0.037, 0.021)	0.602	-	183,990	GLGC
	IVW	−0.003	0.013	(−0.028, 0.022)	0.801	0.396		
	MR Egger	−0.062	0.055	(−0.170, 0.045)	0.459	0.430		
	WM	−0.005	0.004	(−0.012, 0.003)	0.231	-	224,140	UKBB
	IVW	−0.002	0.004	(−0.011, 0.006)	0.589	0.162		
	MR Egger	−0.021	0.014	(−0.048, 0.005)	0.361	0.385		
LDL cholesterol	WM	−0.058	0.019	(−0.095, −0.021)	0.002	-	169,960	GLGC
	IVW	−0.100	0.043	(−0.184, −0.015)	0.020	6 × 10^−5^		
	MR Egger	−0.351	0.059	(−0.467, −0.235)	0.106	0.143		
	WM	−0.089	0.014	(−0.116, −0.062)	5.27 × 10^−11^	-	244,476	UKBB
	IVW	−0.083	0.042	(−0.165, −0.001)	0.048	7.6 × 10^−13^		
	MR Egger	−0.279	0.127	(−0.528, −0.031)	0.271	0.356		
Total cholesterol	WM	−0.047	0.019	(−0.085, −0.010)	0.013	-	184,158	GLGC
	IVW	−0.083	0.044	(−0.169, 0.003)	0.060	1.9 × 10^−5^		
	MR Egger	−0.342	0.057	(−0.454, −0.230)	0.106	0.135		
	WM	−0.096	0.018	(−0.132, −0.061)	8.99 × 10^−8^	-	244,950	UKBB
	IVW	−0.090	0.056	(−0.201, 0.020)	0.109	9.8 × 10^−14^		
	MR Egger	−0.359	0.162	(−0.676, −0.042)	0.270	0.336		
Triglycerides	IVW	0.034	0.012	(0.010, 0.059)	0.006	0.958	174,687	GLGC
	WM	0.033	0.013	(0.007, 0.059)	0.014	-		
	MR Egger	0.049	0.053	(−0.055, 0.154)	0.524	0.986		
	WM	0.047	0.012	(0.025, 0.070)	4.2 × 10^−5^	-	244,754	UKBB
	IVW	0.043	0.016	(0.012, 0.074)	0.006	0.043		
	MR Egger	−0.040	0.035	(−0.109, 0.030)	0.463	0.250		
Copper								
HDL cholesterol	IVW	0.004	0.028	(−0.050, 0.058)	0.880	0.116	94,311	GLGC
	IVW	0.002	0.003	(−0.004, 0.008)	0.448	0.885	221,738	UKBB
LDL cholesterol	IVW	−0.048	0.019	(−0.085, −0.011)	0.011	0.785	89,888	GLGC
	IVW	−0.008	0.013	(−0.033, 0.017)	0.543	0.100	241,831	UKBB
Total cholesterol	IVW	−0.043	0.018	(−0.079, −0.007)	0.020	0.823	94,595	GLGC
	IVW	−0.013	0.016	(−0.044, 0.017)	0.388	0.121	242,304	UKBB
Triglycerides	IVW	−0.024	0.020	(−0.063, 0.015)	0.233	0.241	91,013	GLGC
	IVW	−0.008	0.009	(−0.025, 0.008)	0.333	0.874	242,112	UKBB

Note: P-pleiotropy value for IVW methods represents the Cochran’s Q test, while for MR Egger method represents the intercept test; MR, mendelian randomization; IVW, inverse-variance weighted; WM, weight median.

## Supplementary Materials

The following are available online at https://www.mdpi.com/2072-6643/12/10/3174/s1, Figure S1: Genes in the Kyoto Encyclopedia of Genes and Genomes (KEGG) pathway of cholesterol metabolism predicted to carry iron response elements. Predictions with high, medium, and low confidence are shown in yellow, blue, and orange, respectively. Figure S2: Genes in the KEGG pathway of fatty acid degradation predicted to carry iron response elements. Predictions with high, medium, and low confidence are shown in yellow, blue, and orange, respectively. Table S1: STROBE Statement Checklist of items that should be included in reports of observational studies. Table S2: The number of phenotypes and cases considered in each disease category. Table S3: Descriptive characteristics of the UK Biobank participants and the SNPs included in PheWAS analyses. Table S4: The association between the weighted polygenic risk score of each blood mineral and common confounding factors. Table S5: List of shared and unique outcome–exposure pairs exhibiting consistent MR evidence for causal effects. Table S6: Multivariate IVW MR results for iron and copper. Table S7: The results of leave-one-out MR analysis. Table S8: Lists of mineral–outcome relationships based on sex-stratified MR analysis.

## Abbreviations

FDR, false discovery rate; GLGC, Global Lipids Genetics Consortium; ICD, International Classification of Diseases; IVW, inverse-variance weighted; GRS, polygenic risk score; GWAS, genome-wide association study; HDL, high-density lipoprotein cholesterol; LDL, low-density lipoprotein cholesterol; MR, mendelian randomization; NOME, NO Measurement Error; OR, odds ratio; PheWAS, phenome-wide association study; SNP, single nucleotide polymorphism; SD, standard deviation; SIMEX, simulation extrapolation; T2D, type 2 diabetes; TC, total cholesterol; UKBB, UK Biobank; WM, weighted median.

## References

[1] Hunsaker E.W., Franz K.J. (2019). Emerging Opportunities To Manipulate Metal Trafficking for Therapeutic Benefit. Inorg. Chem..

[2] Waldron K.J., Rutherford J.C., Ford D., Robinson N.J. (2009). Metalloproteins and metal sensing. Nat. Cell Biol..

[3] Ferreira C.R., Gahl W.A. (2017). Disorders of metal metabolism. Transl. Sci. Rare Dis..

[4] Camaschella C., Pagani A., Nai A., Silvestri L. (2016). The mutual control of iron and erythropoiesis. Int. J. Lab. Hematol..

[5] Pietrangelo A. (2016). Mechanisms of iron hepatotoxicity. J. Hepatol..

[6] Collins J.F., Prohaska J.R., Knutson M.D. (2010). Metabolic crossroads of iron and copper. Nutr. Rev..

[7] Myint Z.W., Oo T.H., Thein K.Z., Tun A.M., Saeed H. (2018). Copper deficiency anemia: Review article. Ann. Hematol..

[8] Gulec S., Collins J.F. (2014). Molecular Mediators Governing Iron-Copper Interactions. Annu. Rev. Nutr..

[9] Vashchenko G., MacGillivray R.T.A. (2013). Multi-Copper Oxidases and Human Iron Metabolism. Nutrients.

[10] Bao W., Rong Y., Rong S., Liu L. (2012). Dietary iron intake, body iron stores, and the risk of type 2 diabetes: A systematic review and meta-analysis. BMC Med..

[11] Kennish L., Straub R.H., Oh C., Krasnokutsky S., Samuels J., Greenberg J.D., Huang X., Abramson S.B. (2014). Age-dependent ferritin elevations and HFE C282Y mutation as risk factors for symptomatic knee osteoarthritis in males: A longitudinal cohort study. BMC Musculoskelet. Disord..

[12] Lima S.C.V.C., Arrais R.F., Sales C.H., Almeida M.D.G., De Sena K.C.M., Oliveira V.T.L., De Andrade A.S., Pedrosa L.F.C. (2006). Assessment of Copper and Lipid Profile in Obese Children and Adolescents. Biol. Trace Element Res..

[13] Yu L., Yan J., Zhang Q., Lin H., Zhu L., Liu Q., Zhao C. (2020). Association between Serum Ferritin and Blood Lipids: Influence of Diabetes and hs-CRP Levels. J. Diabetes Res..

[14] Li J., Bao W., Zhang T., Zhou Y., Yang H., Jia H., Wang R., Cao Y., Xiao C. (2017). Independent relationship between serum ferritin levels and dyslipidemia in Chinese adults: A population study. PLoS ONE.

[15] Davies N.M., Holmes M.V., Smith G.D. (2018). Reading Mendelian randomisation studies: A guide, glossary, and checklist for clinicians. BMJ.

[16] Smith G.D., Hemani G. (2014). Mendelian randomization: Genetic anchors for causal inference in epidemiological studies. Hum. Mol. Genet..

[17] Gill D., Greco F.D.G., Walker A.P., Srai S.K., Laffan M.A., Minelli C. (2017). The Effect of Iron Status on Risk of Coronary Artery Disease. Arter. Thromb. Vasc. Biol..

[18] Gill D., Monori G., Tzoulaki I., Dehghan A. (2018). Iron Status and Risk of Stroke. Stroke.

[19] Pichler I., Del Greco M.F., Gogele M., Lill C.M., Bertram L., Do C.B., Eriksson N., Foroud T., Myers R.H., Nalls M. (2013). Serum iron levels and the risk of Parkinson disease: A Mendelian randomization study. PLoS Med..

[20] Kodali H.P., Pavilonis B.T., Schooling C.M. (2018). Effects of copper and zinc on ischemic heart disease and myocardial infarction: A Mendelian randomization study. Am. J. Clin. Nutr..

[21] Gill D., Benyamin B., Moore L., Monori G., Zhou A., Koskeridis F., Evangelou E., Laffan M., Walker A.P., Tsilidis K.K. (2019). Associations of genetically determined iron status across the phenome: A mendelian randomization study. PLoS Med..

[22] Cheng W.-W., Zhu Q., Zhang H.-Y. (2019). Mineral Nutrition and the Risk of Chronic Diseases: A Mendelian Randomization Study. Nutrients.

[23] Benyamin B., Esko T., Ried J., Radhakrishnan A., Vermeulen S., Traglia M., Gögele M., Anderson D., Broer L., InterAct Consortium (2014). Novel loci affecting iron homeostasis and their effects in individuals at risk for hemochromatosis. Nat. Commun..

[24] Evans D.M., Zhu G., Dy V., Heath A.C., Madden P.A.F., Kemp J.P., McMahon G., Pourcain B.S., Timpson N.J., Golding J. (2013). Genome-wide association study identifies loci affecting blood copper, selenium and zinc. Hum. Mol. Genet..

[25] Wu P., Gifford A., Meng X., Li X., Campbell H., Varley T., Zhao J., Carroll R., Bastarache L., Denny J.C. (2019). Mapping ICD-10 and ICD-10-CM Codes to Phecodes: Workflow Development and Initial Evaluation. JMIR Med. Informatics.

[26] Burgess S., Butterworth A., Thompson S.G. (2013). Mendelian Randomization Analysis With Multiple Genetic Variants Using Summarized Data. Genet. Epidemiol..

[27] Bowden J., Smith G.D., Haycock P.C., Burgess S. (2016). Consistent Estimation in Mendelian Randomization with Some Invalid Instruments Using a Weighted Median Estimator. Genet. Epidemiol..

[28] Hemani G., Zheng J., Elsworth B., Wade K.H., Haberland V., Baird D., Laurin C., Burgess S., Bowden J., Langdon R. (2018). The MR-Base platform supports systematic causal inference across the human phenome. eLife.

[29] Willer C.J., Schmidt E.M., Sengupta S., Peloso G.M., Gustafsson S., Kanoni S., Ganna A., Chen J., Buchkovich M.L., Global Lipids Genetics Consortium (2013). Discovery and refinement of loci associated with lipid levels. Nat. Genet..

[30] Sanderson E., Smith G.D., Windmeijer F., Bowden J. (2018). An examination of multivariable Mendelian randomization in the single-sample and two-sample summary data settings. Int. J. Epidemiol..

[31] Bowden J., Del Greco M.F., Minelli C., Smith G.D., Sheehan N.A., Thompson J.R. (2016). Assessing the suitability of summary data for two-sample Mendelian randomization analyses using MR-Egger regression: The role of the I2 statistic. Int. J. Epidemiol..

[32] Burgess S., Thompson S.G. (2017). Interpreting findings from Mendelian randomization using the MR-Egger method. Eur. J. Epidemiol..

[33] Kamat M.A., Blackshaw J.A., Young R., Surendran P., Burgess S., Danesh J., Butterworth A.S., Staley J.R. (2019). PhenoScanner V2: An expanded tool for searching human genotype–phenotype associations. Bioinformatics.

[34] Yavorska O., Burgess S. (2017). MendelianRandomization: An R package for performing Mendelian randomization analyses using summarized data. Int. J. Epidemiol..

[35] Verbanck M., Chen C.-Y., Neale B., Do R. (2018). Detection of widespread horizontal pleiotropy in causal relationships inferred from Mendelian randomization between complex traits and diseases. Nat. Genet..

[36] Looker A.C., Dallman P.R., Carroll M.D., Gunter E.W., Johnson C.L. (1997). Prevalence of iron deficiency in the United States. JAMA.

[37] Domellöf M., Lönnerdal B., Dewey K.G., Cohen R.J., Rivera L.L., Hernell O. (2002). Sex differences in iron status during infancy. Pediatrics.

[38] Quinn J.F., Harris C., Kaye J., Lind B., Carter R., Anekonda T., Ralle M. (2011). Gender Effects on Plasma and Brain Copper. Int. J. Alzheimer’s Dis..

[39] Lopes P.A., Santos M.C., Vicente L., Rodrigues M.O., Pavão M.L., Nève J., Viegas-Crespo A.M. (2004). Trace Element Status (Se, Cu, Zn) in Healthy Portuguese Subjects of Lisbon Population: A Reference Study. Biol. Trace Element Res..

[40] Bo S., Durazzo M., Gambino R., Berutti C., Milanesio N., Caropreso A., Gentile L., Cassader M., Cavallo-Perin P., Pagano G. (2008). Associations of Dietary and Serum Copper with Inflammation, Oxidative Stress, and Metabolic Variables in Adults. J. Nutr..

[41] Zang X., Huang H., Zhuang Z., Chen R., Xie Z., Xu C., Xuming M. (2018). The association between serum copper concentrations and cardiovascular disease risk factors in children and adolescents in NHANES. Environ. Sci. Pollut. Res..

[42] Zhu Y., He B., Xiao Y., Chen Y.-J. (2019). Iron metabolism and its association with dyslipidemia risk in children and adolescents: A cross-sectional study. Lipids Health Dis..

[43] Das De S., Krishna S., Jethwa A. (2015). Iron status and its association with coronary heart disease: Systematic review and meta-analysis of prospective studies. Atherosclerosis.

[44] Gill D., Brewer C.F., Monori G., Trégouët D., Franceschini N., Giambartolomei C., Tzoulaki I., Dehghan A. (2019). INVENT Consortium Effects of Genetically Determined Iron Status on Risk of Venous Thromboembolism and Carotid Atherosclerotic Disease: A Mendelian Randomization Study. J. Am. Hear. Assoc..

[45] Ozdemir A., Sevinc C., Selamet U., Turkmen F. (2007). The Relationship Between Iron Deficiency Anemia and Lipid Metabolism in Premenopausal Women. Am. J. Med. Sci..

[46] Wrede C.E., Buettner R., Bollheimer L.C., Schölmerich J., Palitzsch K.-D., Hellerbrand C. (2006). Association between serum ferritin and the insulin resistance syndrome in a representative population. Eur. J. Endocrinol..

[47] Campillos M., Cases I., Hentze M.W., Sanchez M. (2010). SIREs: Searching for iron-responsive elements. Nucleic Acids Res..

[48] Davis M.R., Rendina E., Peterson S.K., Lucas E.A., Smith B.J., Clarke S.L. (2012). Enhanced expression of lipogenic genes may contribute to hyperglycemia and alterations in plasma lipids in response to dietary iron deficiency. Genes Nutr..

[49] DiNicolantonio J.J., Mangan D., O’Keefe J.H. (2018). Copper deficiency may be a leading cause of ischaemic heart disease. Open Hear..

[50] Alarcón-Corredor O.M., Guerrero Y., De Fernández M.R., D’Jesús I., Burguera M., Burguera J.L., Di Bernardo M.L., García M.Y., Alarcón A.O. (2004). Effect of copper supplementation on lipid profile of Venezuelan hyperlipemic patients. Arch. Latinoam. Nutr..

[51] Kim S., Chao P.Y., Allen K.G.D. (1992). Inhibition of elevated hepatic glutathione abolishes copper deficiency cholesterolemia. FASEB J..

[52] Tosco A., Fontanella B., Danise R., Cicatiello L., Grober O.M.V., Ravo M., Weisz A., Marzullo L. (2009). Molecular bases of copper and iron deficiency-associated dyslipidemia: A microarray analysis of the rat intestinal transcriptome. Genes Nutr..

[53] Carr T.P., Lei K.Y. (1989). In Vivo Apoprotein Catabolism of High Density Lipoproteins in Copper-Deficient, Hypercholesterolemic Rats. Exp. Biol. Med..

[54] Lutsenko S., Burkhead J.L. (2013). The Role of Copper as a Modifier of Lipid Metabolism.

[55] Spivak J.L. (2017). Myeloproliferative Neoplasms. N. Engl. J. Med..

[56] Ginzburg Y.Z., Feola M., Zimran E., Varkonyi J., Ganz T., Hoffman R. (2018). Dysregulated iron metabolism in polycythemia vera: Etiology and consequences. Leukemia.

[57] Kucukguven A., Khalil R.A. (2013). Matrix metalloproteinases as potential targets in the venous dilation associated with varicose veins. Curr. Drug Targets.

[58] Fukaya E., Flores A.M., Lindholm D., Gustafsson S., Zanetti D., Ingelsson E., Leeper N.J. (2018). Clinical and Genetic Determinants of Varicose Veins. Circulation.

[59] Zamboni P., Scapoli G., Lanzara V., Izzo M., Fortini P., Legnaro R., Palazzo A., Tognazzo S., Gemmati D. (2005). Serum Iron and Matrix Metalloproteinase-9 Variations in Limbs Affected by Chronic Venous Disease and Venous Leg Ulcers. Dermatol. Surg..

[60] Chiang C.-P., Chang J.Y.-F., Wang Y.-P., Wu Y.-H., Wu Y.-C., Sun A. (2020). Atrophic glossitis: Etiology, serum autoantibodies, anemia, hematinic deficiencies, hyperhomocysteinemia, and management. J. Formos. Med Assoc..

[61] Smith G.D. (2004). Mendelian randomization: Prospects, potentials, and limitations. Int. J. Epidemiol..

[62] Pierce B.L., Burgess S. (2013). Efficient Design for Mendelian Randomization Studies: Subsample and 2-Sample Instrumental Variable Estimators. Am. J. Epidemiol..

[63] Bowden J., Smith G.D., Burgess S. (2015). Mendelian randomization with invalid instruments: Effect estimation and bias detection through Egger regression. Int. J. Epidemiol..

