# Supplementary material for: The Causal Effects of Blood Iron and Copper on Lipid Metabolism Diseases: Evidence from Phenome-Wide Mendelian Randomization Study
Source: Nutrients. 2020 Oct 17;12(10):3174. doi: 10.3390/nu12103174 (PMC7603077; doi:10.3390/nu12103174)
Supplement: Supplementary file 1 [file nutrients-12-03174-s001.zip › nutrients-948345-supplmentary/nutrients-948345-FS1.pdf]

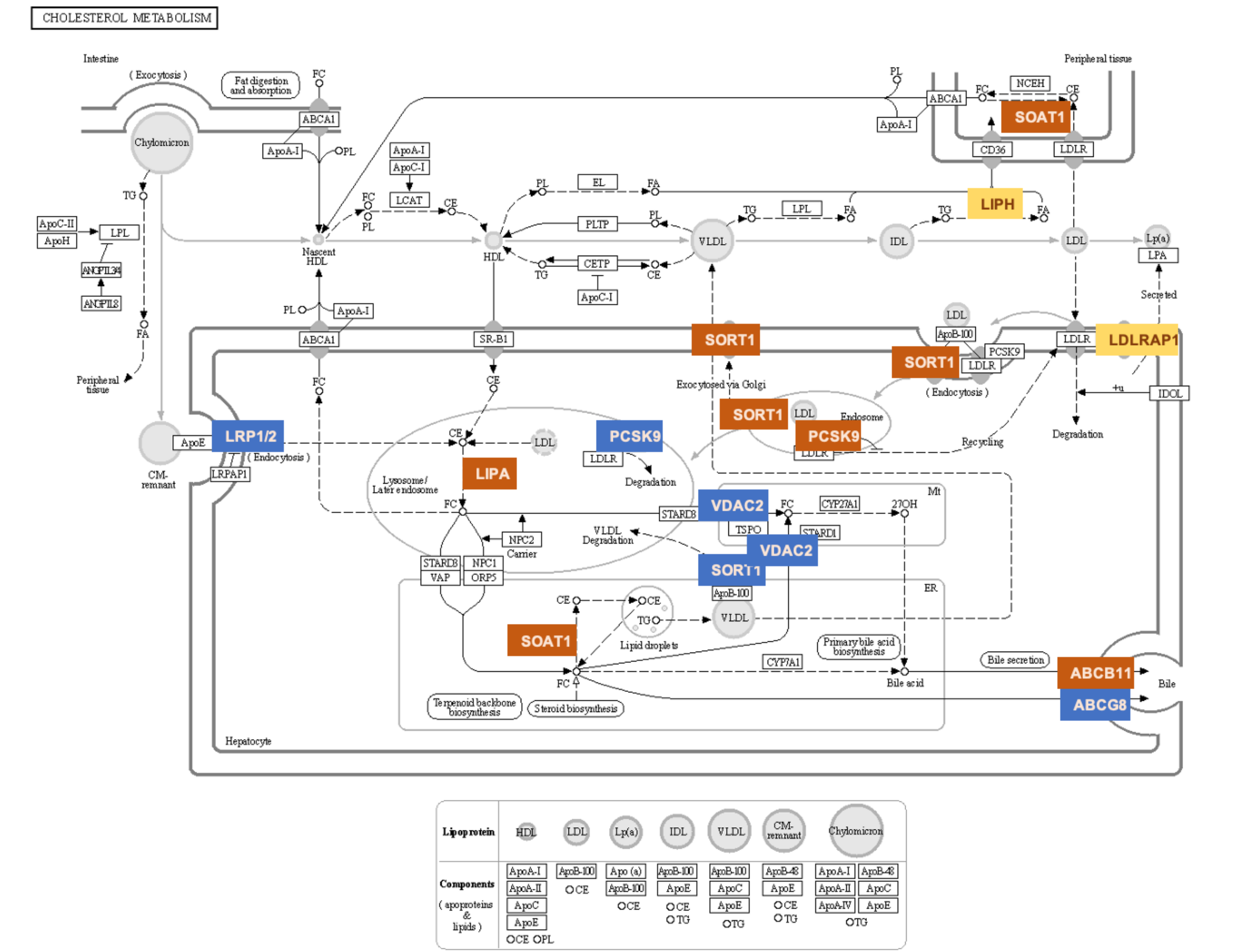
CHOLESTEROL METABOLISM
Intestine
(Exocytosis)
Fat digestion and absorption
FC
ABCA1
Chylomicron
ApoA-I
PL
ApoA-I
ApoC-I
LCAT
FC
PL
CE
TG
ApoC-II
ApoH
LPL
ANGPTL3/4
ANGPTL8
FA
Peripheral tissue
Nascent HDL
HDL
PL
EL
FA
PLTP
PL
CETP
TG
CE
ApoC-I
VLDL
TG
LPL
FA
IDL
TG
LIPH
FA
LDL
Lp(a)
LPA
Secreted
Peripheral tissue
PL
ApoA-I
ABCA1
FC
NCEH
CE
SOAT1
CD36
LDLR
PL
ApoA-I
ABCA1
SR-B1
SORT1
LDL
ApoB-100
PCSK9
SORT1
LDLR
LDLR
LDLRAP1
FC
CE
Exocytosed via Golgi
(Endocytosis)
+u
IDOL
Degradation
SORT1
LDL
Endosome
PCSK9
LDLR
Recycling
CM-remnant
ApoE
LRP1/2
(Endocytosis)
LRPAP1
CE
LDL
PCSK9
LDLR
Degradation
LIPA
Lysosome / Late endosome
FC
STARD3
VDAC2
FC
CYP27A1
27OH
Mt
TSPO
STARD1
NPC2
Carrier
VLDL Degradation
VDAC2
SORT1
STARD3
VAP
NPC1
ORP5
ER
ApoB-100
CE
CE
TG
VLDL
Lipid droplets
SOAT1
Primary bile acid biosynthesis
CYP7A1
Bile secretion
ABCB11
Bile
FC
Bile acid
ABCG8
Terpenoid backbone biosynthesis
Steroid biosynthesis
Hepatocyte
Lipoprotein
HDL
LDL
Lp(a)
IDL
VLDL
CM-remnant
Chylomicron
Components
(apoproteins & lipids)
ApoA-I
ApoA-II
ApoC
ApoE
CE
PL
ApoB-100
CE
Apo (a)
ApoB-100
CE
ApoB-100
ApoE
CE
TG
ApoB-100
ApoC
ApoE
TG
ApoB-48
ApoE
CE
TG
ApoA-I
ApoA-II
ApoA-IV
ApoB-48
ApoC
ApoE
TG
