# Supplementary material for: The Causal Effects of Blood Iron and Copper on Lipid Metabolism Diseases: Evidence from Phenome-Wide Mendelian Randomization Study
Source: Nutrients. 2020 Oct 17;12(10):3174. doi: 10.3390/nu12103174 (PMC7603077; doi:10.3390/nu12103174)
Supplement: Supplementary file 1 [file nutrients-12-03174-s001.zip › nutrients-948345-supplmentary/nutrients-948345-TS.docx]

**Table S1. STROBE Statement,** Checklist of items that should be included in reports of observational studies.

| **Section/Topic** | Item No | Recommendation | Manuscript section and paragraph |
| --- | --- | --- | --- |
| **Title and abstract** | 1 | (*a*) Indicate the study’s design with a commonly used term in the title or the abstract | Title |
|  |  | (*b*) Provide in the abstract an informative and balanced summary of what was done and what was found | Abstract |
| Introduction | | | |
| Background/rationale | 2 | Explain the scientific background and rationale for the investigation being reported | Introduction, paragraphs 1-2 |
| Objectives | 3 | State specific objectives, including any prespecified hypotheses | Introduction, paragraph 3 |
| Methods | | | |
| Study design | 4 | Present key elements of study design early in the paper | Introduction, paragraph 3  Methods, PheWAS and MR analyses sections |
| Setting | 5 | Describe the setting, locations, and relevant dates, including periods of recruitment, exposure, follow-up, and data collection | Methods, Genetic instruments, Study population, PheWAS and MR analyses sections |
| Participants | 6 | (*a*) *Cohort study*—Give the eligibility criteria, and the sources and methods of selection of participants. Describe methods of follow-up  *Case-control study*—Give the eligibility criteria, and the sources and methods of case ascertainment and control selection. Give the rationale for the choice of cases and controls  *Cross-sectional study*—Give the eligibility criteria, and the sources and methods of selection of participants | Methods, PheWAS and MR analyses sections |
|  |  | (*b*) *Cohort study*—For matched studies, give matching criteria and number of exposed and unexposed  *Case-control study*—For matched studies, give matching criteria and the number of controls per case | N/A |
| Variables | 7 | Clearly define all outcomes, exposures, predictors, potential confounders, and effect modifiers. Give diagnostic criteria, if applicable | Methods, Study population, PheWAS and MR analyses sections |
| Data sources/measurement | 8* | For each variable of interest, give sources of data and details of methods of assessment (measurement). Describe comparability of assessment methods if there is more than one group | Methods, Study population, PheWAS and MR analyses sections |
| Bias | 9 | Describe any efforts to address potential sources of bias | Methods, Sensitivity analyses section |
| Study size | 10 | Explain how the study size was arrived at | Methods, PheWAS section |
| Quantitative variables | 11 | Explain how quantitative variables were handled in the analyses. If applicable, describe which groupings were chosen and why | Methods, PheWAS and MR analyses sections |
| Statistical methods | 12 | (*a*) Describe all statistical methods, including those used to control for confounding | Methods, PheWAS and MR analyses and Sensitivity analyses sections |
|  |  | (*b*) Describe any methods used to examine subgroups and interactions | Methods, Sensitivity analyses section |
|  |  | (*c*) Explain how missing data were addressed | N/A |
|  |  | (*d*) *Cohort study*—If applicable, explain how loss to follow-up was addressed  *Case-control study*—If applicable, explain how matching of cases and controls was addressed  *Cross-sectional study*—If applicable, describe analytical methods taking account of sampling strategy | Methods, PheWAS and MR analyses sections |
|  |  | (*e*) Describe any sensitivity analyses | Methods, Sensitivity analyses section |

| **Section/Topic** | Item No | Recommendation | Manuscript section and paragraph |
| --- | --- | --- | --- |
| Results | | | |
| Participants | 13* | (a) Report numbers of individuals at each stage of study—eg numbers potentially eligible, examined for eligibility, confirmed eligible, included in the study, completing follow-up, and analysed | Results, paragraph 1, figure1, Table 2, tableS4-S5 |
|  |  | (b) Give reasons for non-participation at each stage | N/A |
|  |  | (c) Consider use of a flow diagram | Fig 1 |
| Descriptive data | 14* | (a) Give characteristics of study participants (eg demographic, clinical, social) and information on exposures and potential confounders | S2 Table |
|  |  | (b) Indicate number of participants with missing data for each variable of interest | N/A |
|  |  | (c) *Cohort study*—Summarise follow-up time (eg, average and total amount) | N/A |
| Outcome data | 15* | *Cohort study*—Report numbers of outcome events or summary measures over time | S4-S5 Table |
|  |  | *Case-control study—*Report numbers in each exposure category, or summary measures of exposure | S1 Table |
|  |  | *Cross-sectional study—*Report numbers of outcome events or summary measures | S3-S7 table |
| Main results | 16 | (*a*) Give unadjusted estimates and, if applicable, confounder-adjusted estimates and their precision (eg, 95% confidence interval). Make clear which confounders were adjusted for and why they were included | Results, paragraphs 3-8, S3-S7 Table, Figs 2-3 |
|  |  | (*b*) Report category boundaries when continuous variables were categorized | N/A |
|  |  | (*c*) If relevant, consider translating estimates of relative risk into absolute risk for a meaningful time period | N/A |
| Other analyses | 17 | Report other analyses done—eg analyses of subgroups and interactions, and sensitivity analyses | Results, paragraphs 8-10, S4, S5, S6, S7 Tables |
| Discussion | | | |
| Key results | 18 | Summarise key results with reference to study objectives | Discussion, paragraphs 1 |
| Limitations | 19 | Discuss limitations of the study, taking into account sources of potential bias or imprecision. Discuss both direction and magnitude of any potential bias | Discussion, paragraph 5 |
| Interpretation | 20 | Give a cautious overall interpretation of results considering objectives, limitations, multiplicity of analyses, results from similar studies, and other relevant evidence | Discussion, paragraphs 2-5 |
| Generalisability | 21 | Discuss the generalisability (external validity) of the study results | Discussion, paragraph 5 |
| Other Information | | | |
| Funding | 22 | Give the source of funding and the role of the funders for the present study and, if applicable, for the original study on which the present article is based | Funding section |

**Give information separately for cases and controls in case-control studies and, if applicable, for exposed and unexposed groups in cohort and cross-sectional studies.*

**Note:** An Explanation and Elaboration article discusses each checklist item and gives methodological background and published examples of transparent reporting. The STROBE checklist is best used in conjunction with this article (freely available on the Web sites of PLoS Medicine at http://www.plosmedicine.org/, Annals of Internal Medicine at http://www.annals.org/, and Epidemiology at http://www.epidem.com/). Information on the STROBE Initiative is available at [www.strobe-statement.org](http://www.strobe-statement.org).

**Table S2.** The number of phenotypes and cases considered in each disease category.

| **Disease category** | **Iron** | | | | **Copper** | | | |
| --- | --- | --- | --- | --- | --- | --- | --- | --- |
|  | **phenotypes** | **minimum** | **median** | **maximum** | **phenotype** | **minimum** | **median** | **maximum** |
| **Circulatory system** | 103 | 201 | 1267 | 67105 | 102 | 221 | 1277 | 66427 |
| **Congenital anomalies** | 20 | 206 | 372 | 1174 | 20 | 208 | 368 | 1174 |
| **Dermatologic** | 47 | 204 | 777 | 7147 | 46 | 223 | 805 | 7040 |
| **Digestive** | 114 | 216 | 1612 | 30754 | 114 | 218 | 1594 | 30429 |
| **Endocrine/metabolic** | 61 | 205 | 783 | 31283 | 60 | 202 | 785 | 31010 |
| **Genitourinary** | 50 | 212 | 1213 | 12603 | 50 | 210 | 1211 | 12474 |
| **Hematopoietic** | 27 | 208 | 666 | 10828 | 27 | 203 | 650 | 10686 |
| **Infectious diseases** | 26 | 218 | 1225 | 7362 | 26 | 216 | 1214 | 7281 |
| **Injuries & poisonings** | 42 | 250 | 808 | 16374 | 42 | 242 | 800 | 16155 |
| **Mental disorders** | 35 | 237 | 680 | 11722 | 35 | 234 | 675 | 11581 |
| **Musculoskeletal** | 69 | 204 | 912 | 35601 | 69 | 203 | 908 | 35287 |
| **Neoplasms** | 79 | 208 | 865 | 18498 | 79 | 208 | 857 | 18290 |
| **Neurological** | 37 | 208 | 472 | 10040 | 37 | 209 | 466 | 9944 |
| **Respiratory** | 52 | 230 | 1608 | 9818 | 52 | 220 | 1594 | 9692 |
| **Sense organs** | 68 | 200 | 636 | 18654 | 68 | 203 | 628 | 18471 |
| **Symptoms** | 16 | 323 | 1435 | 15063 | 16 | 323 | 1430 | 14902 |

**Table S3.** Descriptive characteristics of the UK Biobank participants (N = 310, 999) and genotype counts of the SNPs included in PheWAS analyses.

| **Characteristics** |  |  |
| --- | --- | --- |
| Age,years(SD) | 56.86(8.0) |  |
| Sex,female(%) | 53.50% |  |
| BMI(SD) | 27.39(4.75) |  |
| SBP | 82.14(10.03) | |
| DBP | 138.33(18.47) | |
| **Genotype counts** |  |  |
| rs1799945 | 304151 |  |
| GG | 6892 | 2.27% |
| GC | 78204 | 25.71% |
| CC | 219055 | 72.02% |
| Hardy-Weinberg test *P-value* | 0.6284714 |  |
|  |  |  |
| rs1800561 | 304151 |  |
| AA | 1922 | 0.63% |
| AG | 43188 | 14.20% |
| GG | 259041 | 85.17% |
| Hardy-Weinberg test *P-value* | 0.9916784 |  |
|  |  |  |
| rs855791 | 304151 |  |
| GG | 95993 | 31.56% |
| GA | 149412 | 49.12% |
| AA | 58746 | 19.31% |
| Hardy-Weinberg test *P-value* | 0.8412966 |  |
|  |  |  |
|  |  |  |
| rs1175550 | 300795 |  |
| AA | 179878 | 59.80% |
| AG | 105630 | 35.12% |
| GG | 15287 | 5.08% |
| Hardy-Weinberg test *P-value* | 0.8297927 |  |
|  |  |  |
| rs2769264 | 300795 |  |
| TT | 199885 | 66.45% |
| TG | 90675 | 30.15% |
| GG | 10235 | 3.40% |
| Hardy-Weinberg test *P-value* | 0.3027299 |  |

**Table S4.** The association between the weighted GRS of each blood mineral and common confounding factors.

|  | Iron GRS |  |  | copper GRS | |  |
| --- | --- | --- | --- | --- | --- | --- |
| **Continuous** | Beta | se | p_value | Beta | se | p_value |
| Variable |  |  |  |  |  |  |
| age | -0.14 | 0.07 | 5.84E-02 | 0.03 | 0.07 | 6.95E-01 |
| BMI | 0.00 | 0.04 | 9.61E-01 | 0.14 | 0.04 | 9.12E-04 |
| PC1 | -0.08 | 0.01 | 3.56E-08 | -0.02 | 0.01 | 2.80E-01 |
| PC2 | 0.00 | 0.01 | 8.08E-01 | -0.01 | 0.01 | 2.91E-01 |
| PC3 | -0.09 | 0.01 | 1.14E-09 | -0.03 | 0.01 | 4.18E-02 |
| PC4 | 0.67 | 0.03 | 2.50E-131 | 0.04 | 0.03 | 1.05E-01 |
| PC5 | 1.91 | 0.06 | 6.84E-212 | -0.07 | 0.06 | 2.36E-01 |
| PC6 | -0.02 | 0.02 | 1.92E-01 | -0.04 | 0.01 | 9.54E-03 |
| PC7 | -0.08 | 0.02 | 1.40E-06 | -0.05 | 0.02 | 1.19E-03 |
| PC8 | 0.08 | 0.02 | 8.30E-06 | -0.07 | 0.02 | 3.90E-05 |
| PC9 | 0.32 | 0.04 | 1.40E-13 | 0.25 | 0.04 | 1.10E-09 |
| PC10 | -0.03 | 0.02 | 1.20E-01 | 0.08 | 0.02 | 8.30E-05 |
| Categorical | F_value | p_value |  | F_value | p_value |  |
| variable |  |  |  |  |  |  |
| Sex | 1.0042 | 0.4185 |  | 1.0083 | 1.09E-01 |  |

Note: GRS: genetic risk score; se, standard error.

**Table S5.** List of shared and unique outcome-exposure pairs exhibiting consistent MR evidence for causal effects.

| **Type** | **exposure** | **outcome** | **method** | **I^2^GX** | **unadjusted** | | | | | **SIMEX adjusted model** | | | |
| --- | --- | --- | --- | --- | --- | --- | --- | --- | --- | --- | --- | --- | --- |
|  |  |  |  |  | **OR** | **upper  95% CI** | **lower  95% CI** | **p value** | **P- pleiotropy** | **OR** | **upper  95% CI** | **lower  95% CI** | **p value** |
| Unique | Fe | Acquired foot deformities | IVW |  | 1.21 | 1.09 | 1.35 | 4.95E-04 | 0.839 | 1.21 | 1.162 | 1.272 | 0.014 |
| Unique | Fe | Acquired foot deformities | MR Egger | 0.9093 | 1.32 | 0.85 | 2.03 | 4.31E-01 | 0.767 | 1.29 | 1.103 | 1.519 | 0.195 |
| Unique | Fe | Acquired foot deformities | WM |  | 1.23 | 1.08 | 1.39 | 1.28E-03 | -- |  |  |  |  |
| Shared | Fe | Disorders of lipoid metabolism | IVW |  | 0.9 | 0.85 | 0.96 | 6.61E-04 | 0.770 | 0.90 | 0.875 | 0.930 | 0.022 |
| Shared | Cu | Disorders of lipoid metabolism | IVW |  | 0.92 | 0.87 | 0.98 | 4.94E-03 | 0.809 | 0.92 | 0.905 | 0.937 | 0.070 |
| Shared | Fe | Disorders of lipoid metabolism | MR Egger | 0.9128 | 0.95 | 0.75 | 1.21 | 7.49E-01 | 0.736 | 0.94 | 0.851 | 1.037 | 0.432 |
| Shared | Fe | Disorders of lipoid metabolism | WM |  | 0.91 | 0.85 | 0.97 | 6.25E-03 | -- |  |  |  |  |
| Unique | Fe | Glossitis | IVW |  | 3.47 | 1.99 | 6.07 | 1.25E-05 | 0.996 | 3.47 | 3.346 | 3.595 | 2.16E-04 |
| Unique | Fe | Glossitis | MR Egger | 0.9152 | 3.3 | 0.39 | 7.56 | 4.69E-01 | 0.968 | 3.31 | 2.779 | 3.948 | 0.048 |
| Unique | Fe | Glossitis | WM |  | 3.45 | 1.84 | 6.45 | 1.08E-04 | -- |  |  |  |  |
| Shared | Fe | Hypercholesterolemia | IVW |  | 0.9 | 0.84 | 0.95 | 5.34E-04 | 0.629 | 0.89 | 0.859 | 0.935 | 0.037 |
| Shared | Cu | Hypercholesterolemia | IVW |  | 0.93 | 0.88 | 0.99 | 2.17E-02 | 0.970 | 0.93 | 0.925 | 0.940 | 0.037 |
| Shared | Fe | Hypercholesterolemia | MR Egger | 0.9124 | 0.95 | 0.74 | 1.22 | 7.52E-01 | 0.725 | 0.93 | 0.800 | 1.086 | 0.532 |
| Shared | Fe | Hypercholesterolemia | WM |  | 0.9 | 0.84 | 0.97 | 4.34E-03 | -- |  |  |  |  |
| Shared | Fe | Hyperlipidemia | IVW |  | 0.9 | 0.85 | 0.96 | 6.44E-04 | 0.743 | 0.90 | 0.872 | 0.931 | 0.025 |
| Shared | Cu | Hyperlipidemia | IVW |  | 0.92 | 0.87 | 0.98 | 5.51E-03 | 0.790 | 0.92 | 0.904 | 0.939 | 0.075 |
| Shared | Fe | Hyperlipidemia | MR Egger | 0.9129 | 0.95 | 0.75 | 1.22 | 7.72E-01 | 0.713 | 0.94 | 0.850 | 1.045 | 0.463 |
| Shared | Fe | Hyperlipidemia | WM |  | 0.91 | 0.85 | 0.97 | 6.42E-03 | -- |  |  |  |  |
| Shared | Fe | Iron deficiency anemias | IVW |  | 0.75 | 0.67 | 0.85 | 1.81E-06 | 0.635 | 0.75 | 0.700 | 0.814 | 0.018 |
| Shared | Cu | Iron deficiency anemias | IVW |  | 0.88 | 0.79 | 0.99 | 3.17E-02 | 0.794 | 0.88 | 0.858 | 0.910 | 0.076 |
| Shared | Fe | Iron deficiency anemias | MR Egger | 0.9129 | 0.68 | 0.43 | 1.1 | 3.60E-01 | 0.746 | 0.67 | 0.499 | 0.886 | 0.220 |
| Shared | Fe | Iron deficiency anemias | WM |  | 0.73 | 0.65 | 0.83 | 8.48E-07 | -- |  |  |  |  |
| Unique | Cu | Osteoarthritis; localized | IVW |  | 1.09 | 1.03 | 1.15 | 3.94E-03 | 0.704 | 1.09 | 1.064 | 1.115 | 0.088 |
| Unique | Cu | Osteoarthrosis | IVW |  | 1.07 | 1.02 | 1.13 | 1.04E-02 | 0.984 | 1.07 | 1.052 | 1.221 | 0.010 |
| Unique | Fe | Other anemias | IVW |  | 0.72 | 0.65 | 0.79 | 1.12E-11 | 0.385 | 0.71 | 0.648 | 0.786 | 0.021 |
| Unique | Fe | Other anemias | MR Egger | 0.9168 | 0.83 | 0.53 | 1.31 | 5.70E-01 | 0.630 | 0.79 | 0.575 | 1.103 | 0.401 |
| Unique | Fe | Other anemias | WM |  | 0.73 | 0.65 | 0.82 | 1.16E-07 | -- |  |  |  |  |
| Unique | Fe | Varicose veins | IVW |  | 1.28 | 1.15 | 1.42 | 4.34E-06 | 0.321 | 1.28 | 1.162 | 1.425 | 0.040 |
| Unique | Fe | Varicose veins | MR Egger | 0.9089 | 1.46 | 0.87 | 2.47 | 3.88E-01 | 0.694 | 1.40 | 0.939 | 2.105 | 0.346 |
| Unique | Fe | Varicose veins | WM |  | 1.29 | 1.15 | 1.45 | 1.46E-05 | -- |  |  |  |  |
| Unique | Fe | Varicose veins of lower extremity | IVW |  | 1.29 | 1.15 | 1.45 | 2.27E-05 | 0.254 | 1.29 | 1.155 | 1.456 | 0.048 |
| Unique | Fe | Varicose veins of lower extremity | MR Egger | 0.9069 | 1.42 | 0.75 | 2.67 | 4.73E-01 | 0.810 | 1.34 | 0.829 | 2.177 | 0.442 |
| Unique | Fe | Varicose veins of lower extremity | WM |  | 1.29 | 1.14 | 1.46 | 6.10E-05 | -- |  |  |  |  |

Note: The I^2^ statistic (I^2^GX) is proposed to quantify the strength of NOME violation for a set of instruments used for MR-Egger regression. SIMEX: Simulation Extrapolation. This differs from regular MR regression because it uses the SIMEX method to correct for regression dilution bias. P-pleiotropy value for IVW methods represent test for balanced horizontal pleiotropy, while for MR Egger method represent intercept test for directional pleiotropy. CI, confidence interval; MR, mendelian randomization.

**Table S6.** Multivariate IVW MR results for iron and copper.

|  |  | **Disorder of lipid metabolism** | | | **Hyperlipidemia** | | | **Hypercholesterolemia** | | | **Anemia** | | |
| --- | --- | --- | --- | --- | --- | --- | --- | --- | --- | --- | --- | --- | --- |
|  |  | **OR** | **(95%CI)** | **p Value** | **OR** | **(95%CI)** | **p Value** | **OR** | **(95%CI)** | **p Value** | **OR** | **(95%CI)** | **p Value** |
| PheWAS | Fe | 0.90 | (0.845,0.957) | 0.0008 | 0.893 | (0.844,0.957) | 0.0008 | 0.89 | (0.837,0.952) | 0.0006 | 0.74 | (0.655,0.833) | 7.65E-07 |
| IVW MR | Fe | 0.91 | (0.853,0.962) | 0.0013 | 0.900 | (0.852,0.962) | 0.0012 | 0.90 | (0.846,0.958) | 0.0009 | 0.75 | (0.575,0.847) | 1.90E-06 |
| Multivariate IVW MR | Fe | 0.90 | (0.848,0.950) | <0.001 | 0.894 | (0.848,0.950) | <0.001 | 0.89 | (0.843,0.948) | <0.001 | 0.75 | (0.672,0.838) | <0.001 |
| PheWAS | Cu | 0.91 | (0.856,0.963) | 0.0012 | 0.918 | (0.857,0.964) | 0.0014 | 0.92 | (0.864,0.976) | 0.0058 | 0.88 | (0.789,0.989) | 0.0318 |
| IVW MR | Cu | 0.91 | (0.856,0.963) | 0.0012 | 0.918 | (0.857,0.964) | 0.0014 | 0.92 | (0.864,0.976) | 0.0058 | 0.88 | (0.789,0.989) | 0.0315 |
| Multivariate IVW MR | Cu | 0.91 | (0.862,0.969) | 0.002 | 0.924 | (0.862,0.969) | 0.003 | 0.92 | (0.870,0.981) | 0.01 | 0.89 | (0.799,0.998) | 0.046 |

Note: OR: odds ratio; se, standard error; CI, confidence interval; IVW, inverse-variance weighted; MR, mendelian randomization.

**Table S7.** The results of leave-one-out analysis depicting the relationship between blood iron or copper with lipid metabolism disease with valid SNPs.

| **Exposure** | **outcome** | **SNP** | **beta** | **se** | **OR** | **p value** |
| --- | --- | --- | --- | --- | --- | --- |
| Blood iron | Disorders of lipid metabolism | rs1799945 | -0.11 | 0.03 | 0.90 | 0.001 |
| Blood iron | Disorders of lipid metabolism | rs1800562 | -0.11 | 0.04 | 0.89 | 0.003 |
| Blood iron | Disorders of lipid metabolism | rs855791 | -0.09 | 0.04 | 0.92 | 0.029 |
| Blood iron | Disorders of lipid metabolism | All | -0.10 | 0.03 | 0.90 | 0.001 |
| Blood iron | Hyperlipidemia | rs1799945 | -0.11 | 0.03 | 0.90 | 0.001 |
| Blood iron | Hyperlipidemia | rs1800562 | -0.11 | 0.04 | 0.89 | 0.003 |
| Blood iron | Hyperlipidemia | rs855791 | -0.08 | 0.04 | 0.92 | 0.031 |
| Blood iron | Hyperlipidemia | All | -0.10 | 0.03 | 0.90 | 0.001 |
| Blood iron | Hypercholesterolemia | rs1799945 | -0.12 | 0.04 | 0.89 | 0.001 |
| Blood iron | Hypercholesterolemia | rs1800562 | -0.12 | 0.04 | 0.89 | 0.003 |
| Blood iron | Hypercholesterolemia | rs855791 | -0.08 | 0.04 | 0.92 | 0.037 |
| Blood iron | Hypercholesterolemia | All | -0.11 | 0.03 | 0.90 | 0.001 |
| Blood copper | Disorders of lipid metabolism | rs1175550 | -0.09 | 0.04 | 0.91 | 0.044 |
| Blood copper | Disorders of lipid metabolism | rs2769264 | -0.08 | 0.04 | 0.92 | 0.029 |
| Blood copper | Disorders of lipid metabolism | All | -0.08 | 0.03 | 0.92 | 0.005 |
| Blood copper | Hyperlipidemia | rs1175550 | -0.09 | 0.05 | 0.91 | 0.074 |
| Blood copper | Hyperlipidemia | rs2769264 | -0.08 | 0.04 | 0.93 | 0.032 |
| Blood copper | Hyperlipidemia | All | -0.08 | 0.03 | 0.92 | 0.006 |
| Blood copper | Hypercholesterolemia | rs1175550 | -0.07 | 0.05 | 0.93 | 0.085 |
| Blood copper | Hypercholesterolemia | rs2769264 | -0.07 | 0.03 | 0.93 | 0.051 |
| Blood copper | Hypercholesterolemia | All | -0.07 | 0.03 | 0.93 | 0.022 |

Note: In the "SNP" column, a specific SNP ID refers to the one removed in the leave-one-out analysis, while "All" refers to the IVW analysis with all SNPs.

**Table S8.** Lists of mineral-outcome relationships based on sex-stratified MR analysis.

| **Type** | **exposure** | **Phecode** | **outcome** | **Group** | **beta** | **se** | **OR (95% CI)** | **p value** | **Q p-value** |
| --- | --- | --- | --- | --- | --- | --- | --- | --- | --- |
| male | Fe | 200 | Myeloproliferative disease | neoplasms | 0.38 | 0.68 | 1.46 (0.38, 5.54) | 5.79E-01 | 2.18E-06 |
| female | Fe | 200 | Myeloproliferative disease | neoplasms | 0.79 | 0.24 | 2.20 (1.37, 3.53) | 1.14E-03 | 8.05E-01 |
| combined | Fe | 200 | Myeloproliferative disease | neoplasms | 0.53 | 0.46 | 1.69 (0.69, 4.19) | 2.52E-01 | 6.43E-05 |
| male | Fe | 200.1 | Polycythemia vera | neoplasms | 1.00 | 1.08 | 2.72 (0.33, 22.56) | 3.54E-01 | 9.78E-08 |
| female | Fe | 200.1 | Polycythemia vera | neoplasms | 1.28 | 0.41 | 3.61 (1.61, 8.12) | 1.89E-03 | 5.75E-01 |
| combined | Fe | 200.1 | Polycythemia vera | neoplasms | 1.08 | 0.82 | 2.93 (0.58, 14.67) | 1.90E-01 | 1.63E-06 |
| male | Fe | 289.8 | Polycythemia, secondary | hematopoietic | 0.39 | 0.62 | 1.48 (0.44, 4.98) | 5.24E-01 | 2.69E-02 |
| female | Fe | 289.8 | Polycythemia, secondary | hematopoietic | 1.47 | 0.63 | 4.34 (1.27, 14.88) | 1.95E-02 | 1.91E-01 |
| combined | Fe | 289.8 | Polycythemia, secondary | hematopoietic | 0.71 | 0.61 | 2.03 (0.62, 6.64) | 2.42E-01 | 6.72E-03 |
| male | Fe | 571 | Chronic liver disease and cirrhosis | digestive | 0.39 | 0.16 | 1.47 (1.07, 2.03) | 1.66E-02 | 1.36E-01 |
| female | Fe | 571 | Chronic liver disease and cirrhosis | digestive | -0.15 | 0.24 | 0.86 (0.54, 1.37) | 5.18E-01 | 2.01E-02 |
| combined | Fe | 571 | Chronic liver disease and cirrhosis | digestive | 0.13 | 0.19 | 1.14 (0.78, 1.66) | 5.10E-01 | 4.25E-03 |
| male | Fe | 571.5 | Other chronic nonalcoholic liver disease | digestive | 0.46 | 0.16 | 1.59 (1.16, 2.17) | 3.98E-03 | 2.57E-01 |
| female | Fe | 571.5 | Other chronic nonalcoholic liver disease | digestive | -0.03 | 0.33 | 0.97 (0.51, 1.86) | 9.32E-01 | 2.41E-03 |
| combined | Fe | 571.5 | Other chronic nonalcoholic liver disease | digestive | 0.21 | 0.24 | 1.23 (0.77, 1.97) | 3.77E-01 | 2.14E-03 |
| male | Fe | 715.2 | Ankylosing spondylitis | musculoskeletal | 0.75 | 0.32 | 2.12 (1.13, 4.00) | 2.00E-02 | 3.26E-01 |
| female | Fe | 715.2 | Ankylosing spondylitis | musculoskeletal | 0.80 | 1.07 | 2.22 (0.27, 18.13) | 4.56E-01 | 9.03E-03 |
| combined | Fe | 715.2 | Ankylosing spondylitis | musculoskeletal | 0.74 | 0.52 | 2.09 (0.76, 5.79) | 1.55E-01 | 1.84E-02 |
| male | Fe | 740.9 | Osteoarthrosis NOS | musculoskeletal | 0.24 | 0.10 | 1.27 (1.05, 1.54) | 1.27E-02 | 2.51E-01 |
| female | Fe | 740.9 | Osteoarthrosis NOS | musculoskeletal | 0.09 | 0.14 | 1.09 (0.83, 1.43) | 5.25E-01 | 1.07E-02 |
| combined | Fe | 740.9 | Osteoarthrosis NOS | musculoskeletal | 0.14 | 0.12 | 1.16 (0.91, 1.47) | 2.34E-01 | 3.15E-03 |
| male | Cu | 250 | Diabetes mellitus | endocrine/metabolic | -0.02 | 0.05 | 0.98 (0.89, 1.08) | 7.01E-01 | 3.86E-01 |
| female | Cu | 250 | Diabetes mellitus | endocrine/metabolic | 0.18 | 0.08 | 1.20 (1.02, 1.40) | 2.72E-02 | 1.79E-01 |
| combined | Cu | 250 | Diabetes mellitus | endocrine/metabolic | 0.06 | 0.04 | 1.06 (0.98, 1.15) | 1.21E-01 | 8.56E-01 |
| male | Cu | 250.2 | Type 2 diabetes | endocrine/metabolic | -0.02 | 0.05 | 0.98 (0.88, 1.08) | 6.38E-01 | 3.95E-01 |
| female | Cu | 250.2 | Type 2 diabetes | endocrine/metabolic | 0.17 | 0.08 | 1.19 (1.00, 1.40) | 4.39E-02 | 1.77E-01 |
| combined | Cu | 250.2 | Type 2 diabetes | endocrine/metabolic | 0.05 | 0.04 | 1.05 (0.98, 1.14) | 1.87E-01 | 8.49E-01 |

Note: OR: odds ratio; se, standard error; CI, confidence interval; Q p-value: heterogeneity determined by the Cochran Q test.
