# Supplementary figures and images for: The Causal Effects of Blood Iron and Copper on Lipid Metabolism Diseases: Evidence from Phenome-Wide Mendelian Randomization Study
Source: Nutrients. 2020 Oct 17;12(10):3174. doi: 10.3390/nu12103174 (PMC7603077; doi:10.3390/nu12103174)

# FATTY ACID DEGRADATION

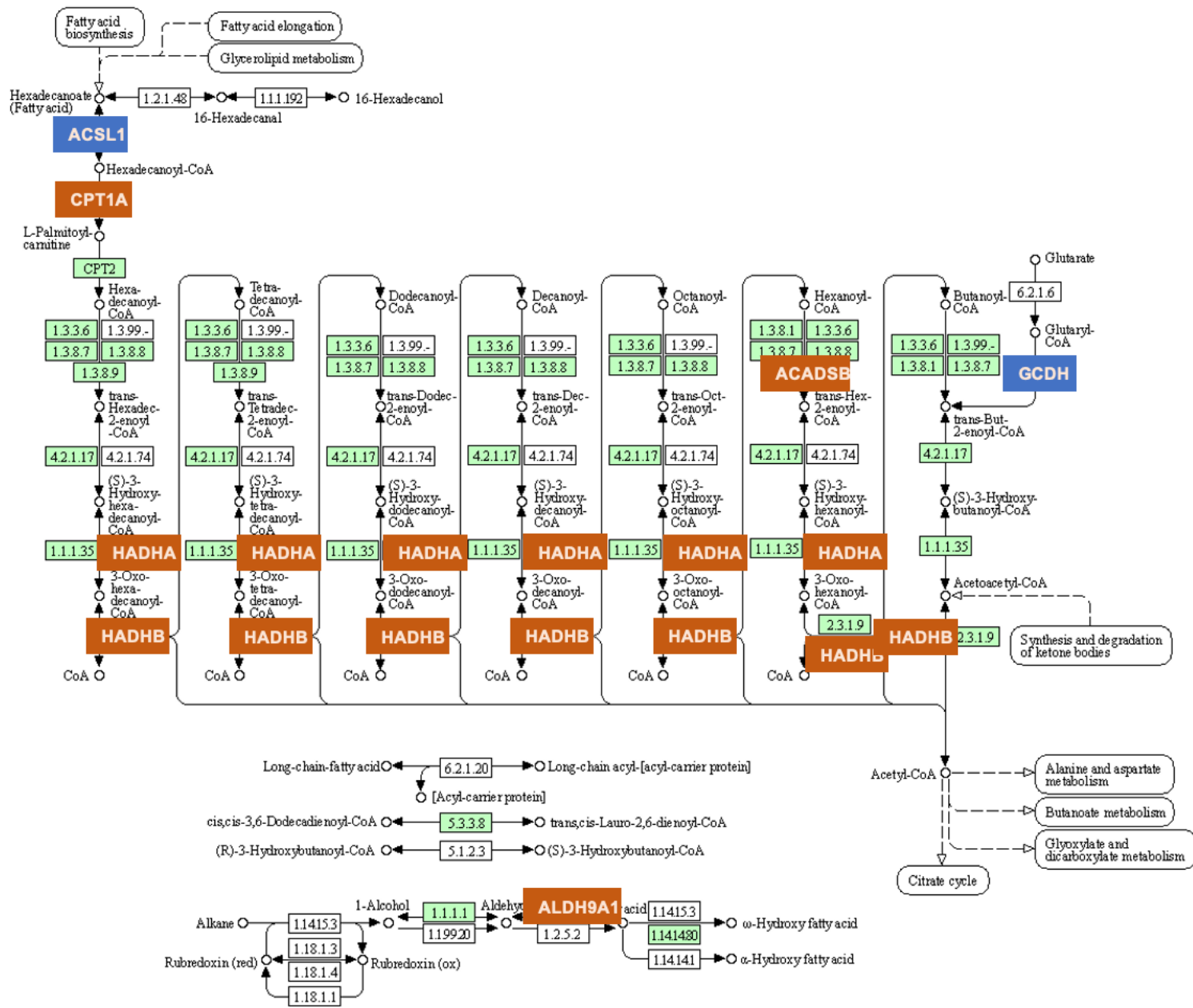

Supplement: Supplementary file 1 [file nutrients-12-03174-s001.zip › nutrients-948345-supplmentary/nutrients-948345-FS2.pdf]
